# Reconstruction of the Aorto–Mitral Curtain in Complex Infective Endocarditis: A Contemporary Narrative Review of the Commando Procedure

**DOI:** 10.31083/RCM49123

**Published:** 2026-08-20

**Authors:** Yilin Lai, Junnan Zheng, Yu Han, Liang Ma, Yiming Ni, Haige Zhao

**Affiliations:** ^1^School of Medicine, Zhejiang University, 310003 Hangzhou, Zhejiang, China; ^2^Department of Cardiovascular Surgery, The First Affiliated Hospital, School of Medicine, Zhejiang University, 310003 Hangzhou, Zhejiang, China

**Keywords:** endocarditis, heart valve diseases, aortic valve, mitral valve, cardiac surgical procedures, aorto-mitral curtain reconstruction

## Abstract

Infective endocarditis (IE) remains a life-threatening condition, particularly in complex cases with destruction of the aorto–mitral curtain (AMC), also known as the intervalvular fibrous body (IVFB). Multiple factors have contributed to an increased incidence of IE and more extensive destruction of the valvular apparatus, necessitating more aggressive surgical intervention. Thus, this study aimed to evaluate the role and outcomes of the Commando procedure in managing IE involving the AMC. A narrative literature review was conducted to provide a comprehensive overview of the Commando procedure in IE involving the AMC, focusing on recent trends, indications, operative strategies, and outcomes. The keywords “infective endocarditis”, “Commando procedure”, “aorto–mitral curtain”, “intervalvular fibrous body”, and “double-valve replacement” were searched in PubMed, Web of Science, and Google Scholar. Only English language studies were included. Inclusion criteria comprised studies involving IE patients who underwent the Commando procedure or any associated modified version; studies reporting conventional double-valve replacement (DVR) performed for IE without AMC involvement were also included for comparison. Exclusion criteria included patients who underwent the Commando procedure for non-infection indications, such as small annuli or calcification. Commando surgery was associated with a 30-day mortality of 9–32%, with 1-, 5-, and 10-year survival rates of 55.4–92.9%, 37.7–68%, and 37–48%, respectively. High-risk factors included infection by specific microorganisms and the need for emergency surgery. The Hemi–Commando procedure demonstrated lower early mortality (8–13.6%), higher 1-year survival (77.5–91%), and 92.3% freedom from reoperation at 1–3 years. Compared with DVR, the Commando procedure had higher operative mortality but similar long-term survival after risk adjustment. Despite the associated high perioperative risks, the Commando surgical procedure remains critical for managing complex IE with AMC destruction. Modified techniques may balance infection control and functional preservation, potentially improving survival in selected patients. Future research should focus on standardized surgical protocols, long-term durability, and multidisciplinary collaboration to optimize outcomes.

## 1. Introduction

Infective endocarditis (IE) remains a life-threatening disease with high morbidity and mortality, especially in complex cases involving destruction of the aorto-mitral curtain (AMC), also known as the intervalvular fibrous body (IVFB). Surgical reconstruction of this region often requires advanced techniques such as the Commando procedure and its modified techniques. Although increasing clinical experience with the Commando procedure and its modified techniques has been reported in recent years, the existing evidence remains limited. Most studies consist of small retrospective series, and there is still a lack of standardized surgical strategies, clearly defined operative indications, and comprehensive evaluation of clinical outcomes. In addition, available reports often include limited patient numbers, relatively short follow-up durations, and insufficient discussion of perioperative management, highlighting the need for a more systematic synthesis of the current evidence. In this review, we focus on the application of the Commando procedure in IE, highlighting its indications, technical considerations, clinical outcomes, and the future research directions, with the aim of providing an overview of current evidence and informing clinical decision-making.

## 2. Infective Endocarditis

IE has an annual incidence of 15 to 80 cases per million people and an in-hospital mortality of up to 20–30% [1,2,3,4]. IE increasingly affects older populations and males, also individuals with prosthetic valves, cardiac devices, or those with underlying comorbidities [4,5].

Surgical intervention is required in 25%–50% of IE patients to address life-threatening complications such as heart failure, uncontrolled infection, or systemic embolism. The timing and strategy of surgery are critical and are guided by the severity of valve dysfunction, extent of infection, and risk of embolic events [1,4,6,7].

The shifting microbiological landscape influences both the timing and type of surgical intervention. *Staphylococcus aureus* is now the predominant pathogen, mainly due to the rising prevalence of intravenous drug use, prosthetic devices, and healthcare-associated infections, which are closely associated with poor outcomes and often requires more aggressive surgery [8].

In advanced IE, the infection extends beyond the valve leaflets to involve the annulus, multiple valves, or even perivalvular structures, resulting in increased surgical challenges. Left-sided IE, particularly involving the aortic and mitral valves, accounts for the majority of cases, and double-valve IE represents about 15–20%, often leading to severe hemodynamic compromise, which often needs extensive debridement and complex reconstruction to eradicate infection and restore cardiac function and is associated with higher mortality [9,10,11]. In these situations, more complex reconstructive procedures, including the Commando procedure, may be required to restore structural integrity and optimize outcomes.

### 2.1 Valvular Surgery in IE

#### 2.1.1 Indications

Surgery is crucial for left-sided IE, to address life-threatening complications and improve survival. The decision to operate in acute IE depends on three primary indications: heart failure, uncontrolled infection, and prevention of systemic embolism [6].

Indications for surgery in tricuspid valve IE are less well-defined. Common indications include large vegetations causing septic pulmonary embolism, severe tricuspid regurgitation, and lack of response to medical therapy [7,12].

In cases involving multiple valves, surgery is generally guided by the left-sided IE [13].

##### 2.1.1.1 Heart Failure

Heart failure is the most frequent complication in 19–73% of native valve infective endocarditis cases and also the main indication for emergency and urgent surgery [4,14,15,16]. Severe valvular dysfunction, including perforation, regurgitation, or invasive structural damage leading to hemodynamic instability, requires immediate intervention.

Heart failure is independently associated with poor in-hospital and 1-year survival [17,18]. Surgery is the only effective approach that may improve survival, especially in New York Heart Association (NYHA) functional class III–IV patients [14].

##### 2.1.1.2 Uncontrolled Infection

Surgical intervention is required for locally uncontrolled infections, such as annular or aortic abscesses, fistulas, enlarging vegetations, or persistent bacteremia despite appropriate antibiotic therapy. Infections caused by multidrug-resistant pathogens or with systemic spread (e.g., septic emboli) also require surgery [4].

##### 2.1.1.3 Prevention of Systemic Embolism

Embolic complications occur in 13%–49% of IE cases. Life-threatening events, such as strokes, occur in 20%–40% [19,20]. Early surgery is recommended for patients with vegetations ≥10 mm in length, particularly those with a history of embolic events despite appropriate antibiotic therapy. Asymptomatic patients with large vegetations and high embolism risk can also benefit from early surgery [4].

#### 2.1.2 Timing of Surgery

The timing of surgery is categorized based on the severity of the condition and includes emergency (<24 hours), urgent (3–5 days), or non-urgent (within the same hospitalization) [21]. Emergency surgery is required for cardiogenic shock, acute valve obstruction, or invasive infections with hemodynamic failure [3]. Urgent surgery should be performed within 3–5 days for patients with persistent sepsis or recurrent embolic events. Stable patients, such as those with large vegetations but no embolic events, are recommended for elective surgery. Delayed surgery (up to 4 weeks) is advised for patients with large cerebral emboli (>2 cm), intracranial hemorrhage, or severe neurological deficits to reduce perioperative mortality [22,23]. Early surgery is also considered for small embolic lesions (<2 cm) and those without hemorrhage or major neurologic deficits [24].

There is also a trend towards early surgery if valve repair is possible instead of replacement [6].

For complex IE cases, early surgery can improve 1-year survival by 20% [4]. Therefore, multidisciplinary management is essential to control perioperative risks and evaluate surgical indications and long-term prognosis.

#### 2.1.3 Surgical Approach and Techniques

Surgery for IE aims to remove the infected tissue and reconstruct the anatomical structure and hemodynamic function. The decision to repair or replace affected heart valves depends on the extent of damage, urgency, and patient characteristics [4]. In mitral valve IE, leaflet perforations that preserve the free edge and chordae tendineae can usually be repaired. However, though more complex cases involving the annuli, leaflet-free edge, and/or chordae tendineae may allow repair, their long-term feasibility and effectiveness remain uncertain [25]. Valve preservation in acute IE is only attempted if infection can be completely eradicated and the repair is durable. In pediatric patients, valve repair is preferred due to limited options for valve replacement [4]. For right-sided IE, valve replacement is considered only when repair is not feasible following debridement of all infected tissue.

Aortic valve IE typically requires valve replacement. Repair is uncommon in acute IE but may be considered for isolated aortic valve regurgitation after recovery from IE. Mild aortic annular damage, such as very limited abscesses or pseudoaneurysms, can still be treated with conventional valve replacement, but extensive aortic root abscesses or periannular destruction often necessitate aortic root replacement [4].

In double-valve IE, mitral valve repair may offer survival benefits over double-valve replacement (DVR), but further studies are still needed. Preventing the spread of infection to multiple valves is crucial to improving outcomes, and valve repair should be prioritized over replacement when possible [9].

Allografts provide advantages in accommodating irregular surfaces, enhancing hemodynamic function and reducing thromboembolic risks [26,27]. Furthermore, allografts and stentless bioprostheses are beneficial for smaller aortic roots and have lower reinfection rates [4].

In cases of infection extending to the AMC, complex reconstruction such as the Commando procedure may be required, given that the Commando procedure is often performed as a salvage operation when alternative surgical options are limited, clear absolute contraindications have not been well defined in the existing literature. Instead, several clinical conditions may limit its feasibility, including profound hemodynamic instability, unreconstructable tissue destruction, and severe comorbidities precluding complex surgery.

## 3. The Commando Techniques

The AMC is a fibrous sheet extending between the left and right trigones, within the continuation between the aortic valve leaflets (left and noncoronary) and the anterior mitral valve leaflet. Aortic and mitral valve replacement with AMC reconstruction was named “Commando” by Bruce Lytle and David Tirone [28,29] and is required in rare cases of extensive aortic root or mitral valve IE patients. The perioperative survival rate is approximately 84% with a 1-year mortality of 20%–30% [4,30,31].

When deciding whether to proceed with Commando surgery, careful consideration must be given to the patient’s overall condition, the extent of valve damage, and the potential high risks associated with the surgery.

### 3.1 The Commando Surgery Technique

Commando surgery is a complex cardiac procedure involving total excision of the aortic valve, mitral valve and AMC, followed by reconstruction using patch materials (e.g., autologous or bovine pericardium, or Dacron) and double valve replacement.

#### 3.1.1 Debridement and Excision

**Fig. 1.**
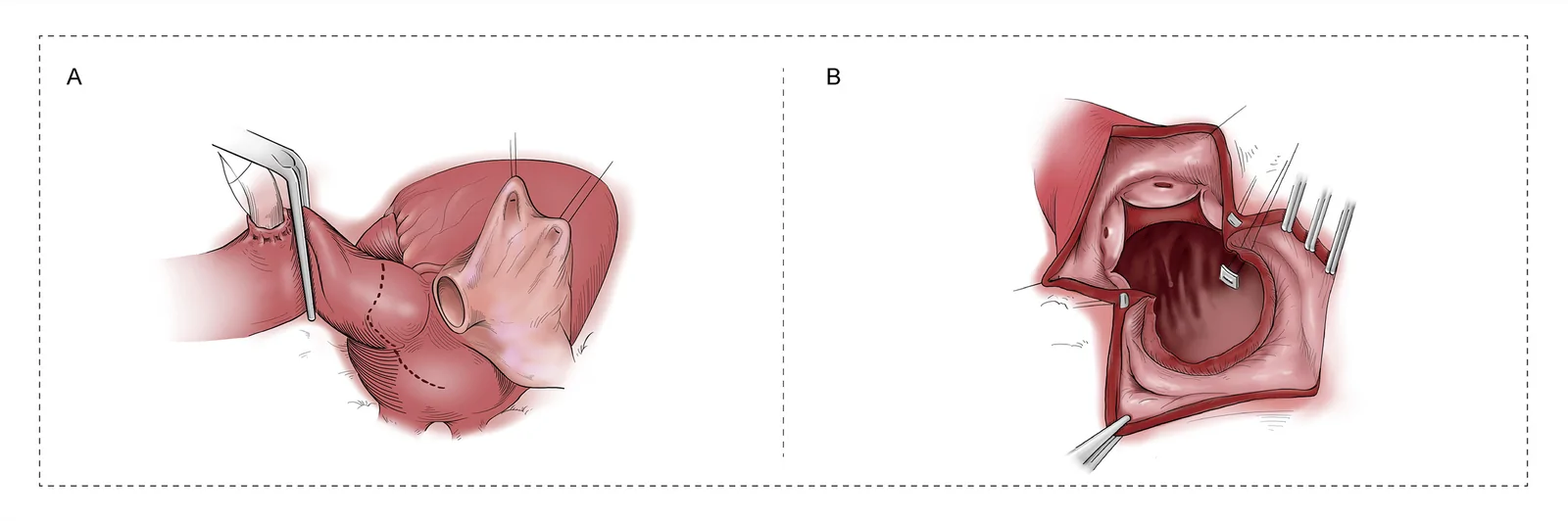
**Total excision of the aortic valve, mitral valve and aorto-mitral curtain (AMC)**. (A) The aortotomy is extended from the noncoronary cusp through the AMC and onto the left atrial roof. The incision starts as a transverse aortotomy and continues obliquely along the noncoronary sinus, extending into the mitral annulus and the dome of the left atrium, dividing the aortic annulus, AMC, and anterior mitral annulus. (B) The infected or nonviable tissue, including the aortic valve, mitral valve and AMC was removed completely.

Radical removal of vegetations, abscesses, and infected or calcified tissue, including the aortic valve, mitral valve, and AMC. An oblique aortotomy is performed and extended through the noncoronary sinus into the AMC, and towards the anterior mitral leaflet while opening the roof of the left atrium (Fig. 1A,B).

#### 3.1.2 Valve Replacement and Reconstruction

Aortic and mitral valves are replaced with mechanical or bioprosthetic prostheses based on patient factors such as age, comorbidities, and the need for long-term anticoagulation. Mitral valve repair may be preferred over replacement in cases of limited valve damage, while aortic valve repair remains challenging and usually requires replacement.

#### 3.1.3 Reconstruction of AMC

**Fig. 2.**
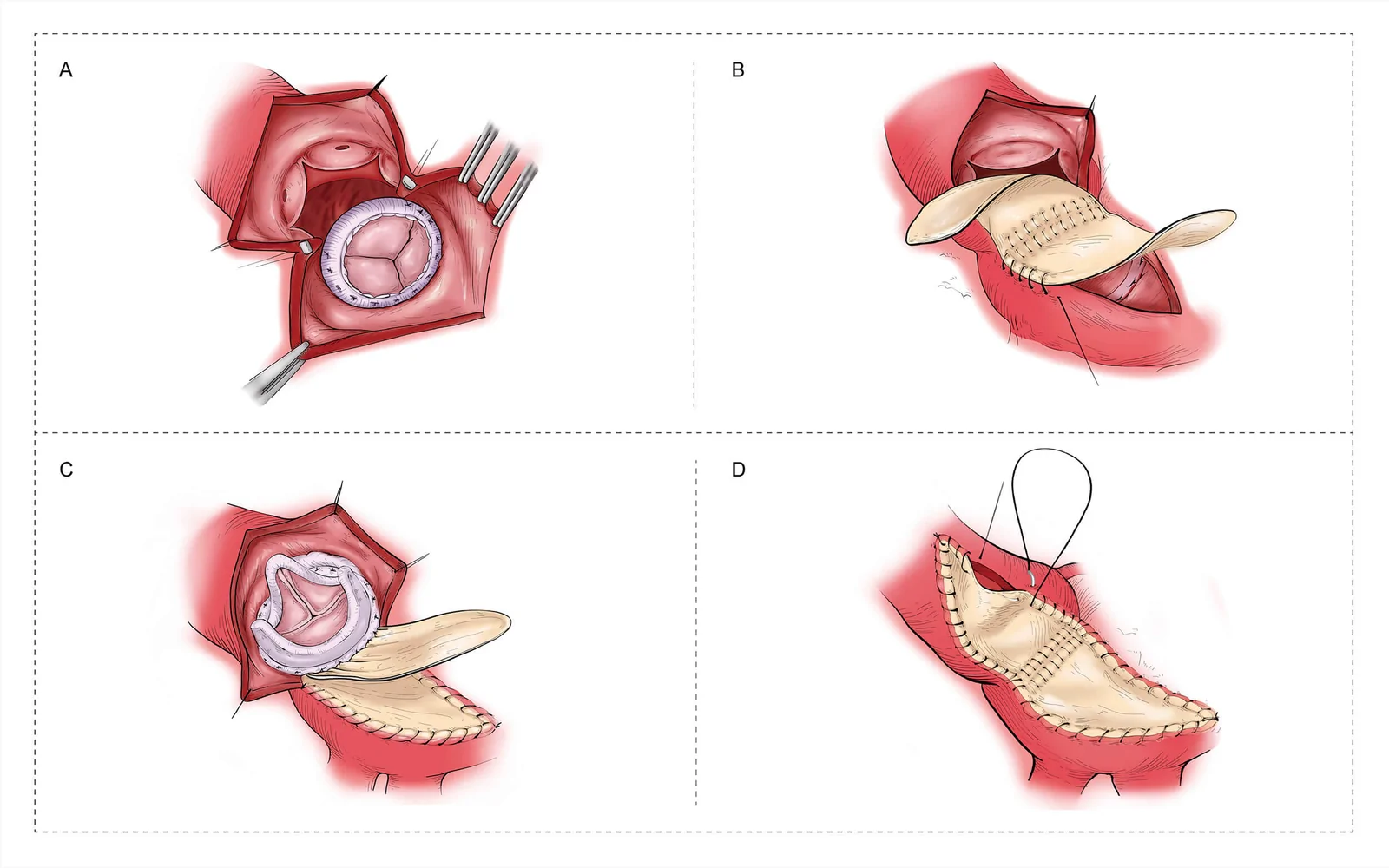
**Double valve replacement and reconstruction of AMC**. (A) An appropriately sized mitral valve prosthesis is secured posteriorly to the mitral annulus and superiorly to the patch. (B) A rhomboid patch is used to reconstruct the aorto-mitral curtain (AMC), left atrial roof and aortic root. The anterior prosthesis mitral annulus is reconstructed with interrupted sutures along the midline of the patch and a limb of the patch is used to close the left atriotomy. (C) An aortic valve prosthesis is secured to the aortic annulus within the right and left coronary sinuses and the noncoronary sewing ring is sutured to the patch. (D) The second limb of the rhomboid patch is used to close the aorta.

Extensive damage requires AMC reconstruction, usually with autologous pericardium is preferred; however, bovine pericardium or synthetic materials are also used. The new AMC length is determined by the distance between the aortic and mitral valve suture lines on the patch, ideally 0.5–1.5 cm to avoid excessive tension that could cause rupture or paravalvular leaks (<1 cm) or patch instability resulting in rocking of the valves (>1.5 cm). The upper edge of the mitral valve is best placed about 1 cm below the aortic valve annulus, with 25%–33% of the valve circumference anchored to the patch [32,33] (Fig. 2A,B,C,D).

In some cases, aortic root replacement, coronary artery bypass grafting, or atrial septal defect repair may be required.

Early postoperative complications include bleeding, sepsis, neurologic events, and conduction disturbances. Late complications include paravalvular leaks, prosthetic valve failure, patch calcification, and pseudoaneurysms.

### 3.2 Hemi-Commando Techniques

Hemi-Commando is a surgical procedure involving aortic valve replacement and AMC reconstruction while preserving the mitral valve, which results in better maintenance of the geometry and function of the left ventricle compared to Commando surgery, especially in high-risk patients or those with multiple comorbidities. Intraoperative echocardiography and meticulous evaluation of the valve after radical removal of the infection guides surgical decisions to ensure adequate attachment and mobility of the free margins of the posterior mitral leaflet, posterior annulus, and anterior mitral leaflet following excision [34].

Compared to Commando surgery, Hemi-Commando balances infection control with functional preservation, reduces procedure complexity, shortens operative time, and has particular importance in patients with high risks.

### 3.3 Modified Commando Techniques

The modified Commando techniques for IE offer innovative approaches to enhance surgical outcomes by tailoring the procedure to the extent of the infection and the specific needs of the patient.

#### 3.3.1 Hinged Ring Technique

**Fig. 3.**
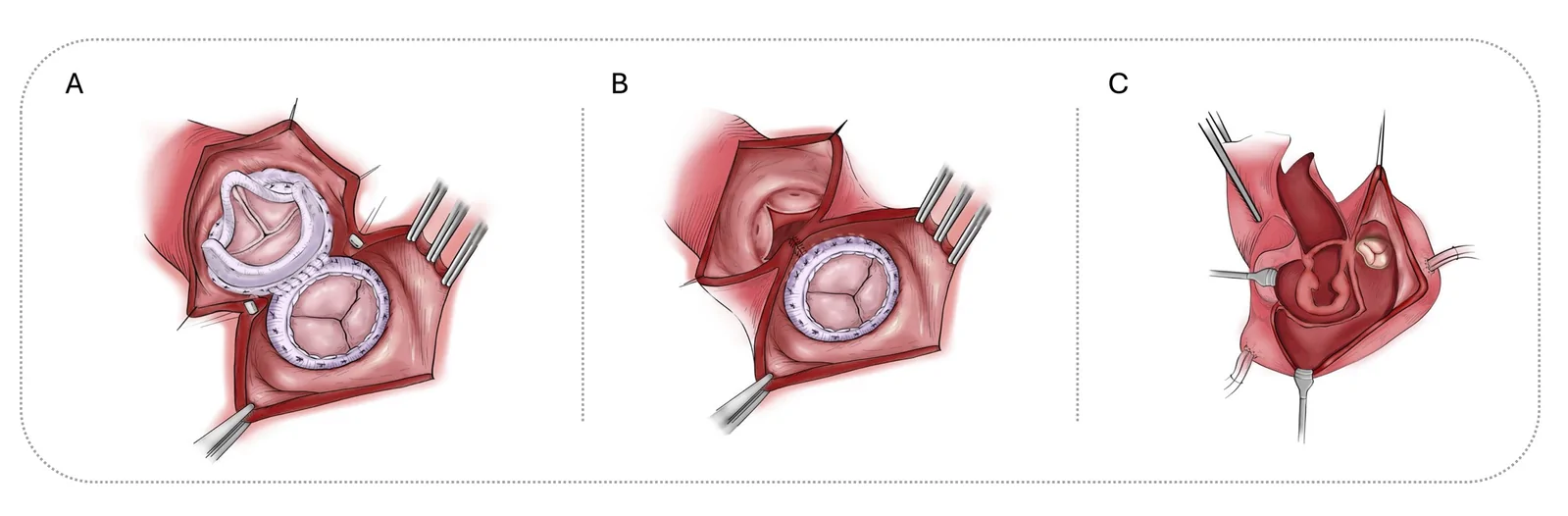
**Some of the modified **C**ommando techniques**. (A) Hinged Ring Technique: The prosthetic aortic valve is anchored to a portion of the sewing ring of the prosthetic mitral valve and the LVOT tissue, effectively reducing the risk of left ventricular outflow tract (LVOT) obstruction. (B) The autologous tissue reconstruction technique: The left atrial wall is utilized to reconstruct the AMC when autologous pericardium is unavailable. (C) Extended Interatrial Approach: The extended interatrial approach involves a combined incision through the right atrium, atrial septum, and left atrium, extending toward the AMC and the transverse aortotomy.

The aortic valve prosthesis is fixed to part of the sewing ring of the prosthetic mitral valve and healthy left ventricular outflow tract (LVOT) tissue, simplifying the procedure and providing a durable repair without LVOT obstruction, avoiding the need for allograft material when the intertrigonal tissue and mitral annulus retain enough structural integrity [35] (Fig. 3A).

#### 3.3.2 Autologous Tissue Reconstruction

In recurrent IE requiring both aortic root and double valve replacement, the left atrium can be used to reconstruct the AMC when the autologous pericardium is unavailable due to extensive adhesions. A smaller-sized mitral valve can reduce AMC tension, but over-sizing may cause patient-prosthesis mismatch or LVOT narrowing. This technique circumvents complications of heterologous grafts, such as bleeding and infection, while utilizing more robust autologous tissue that offers better feasibility for reoperation [36] (Fig. 3B).

#### 3.3.3 Extended Interatrial Approach

A combined incision through the right atrium, atrial septum, and left atrium provides a better surgical field, and the use of an extra patch to repair the right atrium and atrial septum enhances visibility and reduces the tension of IVFB. This approach also reduces the risk of tissue tearing or bleeding. Glutaraldehyde solution is used to treat the remaining tissue surface, which may decrease the rate of postoperative infection [37] (Fig. 3C).

In clinical practice, the distinction between conventional DVR and the Commando procedure is primarily determined by whether the infection involves the AMC. However, no universally accepted quantitative threshold defines the extent of AMC destruction that mandates Commando reconstruction, as current evidence is largely derived from small retrospective series and institutional experience.

Conventional DVR may be feasible when IE involves the aortic and mitral valves but does not extend to the AMC, allowing both annuli to remain structurally intact. In contrast, the Commando procedure becomes necessary when infection extends to the AMC, resulting in destruction and loss of structural continuity between the two valves.

The choice between Hemi-Commando and Commando procedure depends primarily on the extent of mitral involvement: Hemi-Commando may be considered when destruction is largely limited to the anterior mitral leaflet or its base, whereas Commando is typically required when both valvular annuli and the AMC are extensively involved.

Selecting between modified and traditional Commando techniques depends on factors such as the extent of infection, tissue integrity, anatomical exposure requirements, material availability, and the patient’s overall condition. Modified approaches simplify the surgery, reduce cardiopulmonary bypass time, and decrease the incidence of complications, though long-term follow-up is needed to assess the durability and biomaterial safety in infected tissues.

In addition, aggressive pathogens such as fungal organisms or *Staphylococcus aureus*, as well as recurrent prosthetic valve endocarditis, often indicate more destructive disease and may favor a more radical reconstructive approach such as the Commando procedure.

In rare cases of unreconstructable disease or end-stage cardiac failure, heart transplantation may be considered as a salvage option, although experience remains limited. Therefore, surgical decision-making should integrate the extent of anatomical destruction, microbiological characteristics, and hemodynamic status to guide individualized operative strategies.

### 3.4 Perioperative Outcomes

The 30-day mortality ranges from 9% to 32%, and is higher in emergency surgeries or *Staphylococcus aureus* infections. High-risk factors for early death include female sex, age >60, left ventricular ejection fraction (LVEF) <35%, preoperative shock, and concomitant aortic root replacement [38,39,40,41,42,43].

One-year survival after the Commando procedure ranges from 55.4% to 92.9%, two-year from 79.2% to 92.9%, five-year survival 37.7% to 68%, and ten-year survival from 37% to 48% [42,43,44]. For the Hemi-Commando, one-year survival ranges from 77.5% to 91% and three-year survival from 66.4% to 82%, with a freedom-from-reoperation rate of 92.3% at both 1 and 3 years [34]. The Hemi-Commando procedure also shows higher 5- and 7-year survival rates, although longer follow-up is still required [40]. Despite technical complexity, Commando and Hemi-Commando procedures remain viable with acceptable outcomes.

Compared to DVR, Commando surgery has a significantly higher operative mortality and lower survival, consistent with its more demanding technical requirements and the need for more extensive tissue reconstruction [45].

In pediatric patients, a small case series of five patients undergoing the Commando procedure reported no early or late deaths, reoperations, pacemaker implantations, or clinically significant gradients across the mitral or aortic valve during a median follow-up of 29.3 months. Although these results indicate favorable early- to mid-term outcomes, the long-term outcomes of this procedure in pediatric patients remain uncertain because of the small sample size and limited follow-up duration [46].

Most of the evidence is retrospective, focusing on mortality, reoperation rate, and recurrence of IE (Table 1, Ref. [28,34,40,41,43,44,46,47,48,49,50,51,52]).

**Table 1.** **Reported early and late outcomes of Commando, Hemi-Commando and Double-valve Replacement (DVR) for infective endocarditis (IE)**.

	First author	Total patients	Follow-up	Survival				Reoperation		Recurrent IE
Commando	Tirone E. David [28]	43 patients	Mean follow-up 38 ± 29 months	7 operative deaths (16%) 6 late deaths (14%) Actuarial survival 6 years 56% ± 6%						2 patients
	Xuan Jiang [43]	14 patients	Mean follow up 18.9 ± 12.2 months	No late death during follow-up Overall survival 1 year 92.9% 2 years 92.9% 3 years 92.9%				Freedom from reoperation 1 year 100% 2 years 100% 3 years 100%		No recurrent IE
	Hiroo Kinami [46]	5 pediatric patients	Median follow-up 29.3 months (48.0 months)	No early or late deaths				100% freedom from reoperation during follow-up		No recurrent IE
	Markian Bojko [44]	41 patients	The average (standard deviation) follow-up 1.21 (1.86) years	Operative mortality 34.1% 19 follow-up deaths				No late reoperation		
				Overall survival 1 year 55.4% 3 years 50.3% 5 years 37.7%		27 hospital survivors’ overall survival 1 year 87.1% 2 years 87.1% 3 years 79.1%				
	James A. Brown [41]	21 patients	2010 to 2022	Surgical mortality 28%						
	Jose L. Navia [40]	138 (52 Hemi-Commando) patients	Mean follow-up 41 ± 5.9 months	Overall survival 1 year 67% 5 years 48% 10 years 37%				Freedom from reoperation 1 year 87% 5 years 74% 8 years 55%		Freedom from recurrent IE 1 year 90% 5 years 78% 8 years 67%
	Piroze M. Davierwala [49]	127 patients	Mean follow-up 0–136.4 months	Overall survival 3 years 45.3 ± 5.1% 5 years 41.8 ± 5.8%				A total of 9 (7.0%) required reoperations 5-year freedom from reoperation 85.1 ± 5.7%		
	Alberto Forteza-Gil [48]	78 patients	Median follow-up 4.69 years	In-hospital and/or 30-day mortality 14 (17.9%) 5 (16.7%) patients in the ROOT group 9 (18.8%) patients in non-ROOT				2 early reoperations		Reinfection rates ROOT group 1 (3.3%) non-ROOT group 3 (6.2%)
				Overall survival 1 year 76.2% 2 years 76.2% 5 years 67.2%	ROOT group 1 year 79% 2 years 79% 5 years 63%		non-ROOT group 1 year 74% 2 years 74% 5 years 68%			
Hemi-Commando	Tomšic A [50]	35 patients	Median follow-up of 29.8 months	1 year 69.4% 3 years 65.3% 5 years 65.3%				Freedom from reoperation 1 year 84.6% 2 years 78.9% 5 years 51.2%		2 late IE
	Haytham Elgharably [51]	37 patients	Mean follow-up 581 ± 729 days	3 hospital deaths (8%) 2 post-discharge deaths Survival rate 1 year 91% 3 years 82%				1 reoperation (3%)		1 recurrent IE (3%)
	Mateo Marin-Cuartas [34]	22 patients	Median follow-up 12.0 (0.8–36.8) months	30-day mortality 13.6% Survival rate 1 year 77.5% 3 years 66.4%				Freedom from reoperation 1 year 92.3% 3 years 92.3%		
DVR	Paighton C. Miller [47]	211 patients	Median follow-up 2.4 years (0–18.5)	In-hospital mortality 13% (30 deaths (14%) within 30 days) Median overall survival 4.1 years Actuarial survival rate 5 years 45.4% 10 years 36.4%				4 reoperations		3 patients
	Thierry Caus [52]	96 patients two bioprostheses (TBP) group 48 patients two mechanical valves (TMV) group 48 patients	Mean follow-up TBP group 9.6 years TMV group 10.5 years	Survival rate				Freedom from reoperation		
				TBP group 5 years 72.5% ± 6.1% 10 years 43.6% ± 8.3% 15 years 29.7% ± 8.5%		TMV group 5 years 78.9% ± 6.2% 10 years 62.3% ± 7.3% 15 years 35.6% ± 9.3%		TBP group 5 years 83.1% ± 10.2% 10 years 34.4% ± 15.2% 15 years 28.1% ± 18.1%	TMV group 5 years 88% ± 8% 10 years 80.6% ± 12.2% 15 years 76% ± 14.6%	

IE, infective endocarditis; ROOT, patients undergoing full aortic root replacement; non-ROOT, patients without root replacement.

Multiple studies have shown that, the high 5-year mortality is largely driven by early deaths within 90 days, which may be related to the urgency of presentation, poor preoperative status, surgical complexity and its complications [41]. Once passed this period, the postoperative mortality appeared to stabilize. After multivariate risk adjustment, long-term survival following the Commando procedure was not significantly different from that of the Hemi-Commando or DVR groups (Table 2, Ref. [40,45,53]) [41].

**Table 2.** **Different early and late outcomes of Commando, Hemi-Commando and double-valve replacement (DVR) for IE**.

	First author	Total patients	Follow-up	Survival		Reoperation	Recurrent IE
Commando vs Hemi-Commando	Jose L. Navia [40]	138 patients Commando procedure (86 patients) Hemi-Commando procedure (52 patients)	Mean follow-up 41 ± 5.9 months median follow-up 53 months	4 intraoperative deaths in the Commando group no intraoperative deaths in the Hemi-Commando group 28 postoperative in-hospital death (20%) 21 hospital deaths (24%) in the Commando group 7 hospital deaths (14%) in the Hemi-Commando group Overall survival 1 year 67% 5 years 48% 10 years 37%		Freedom from reoperation 1 year 87% 5 years 74% 8 years 55%	Freedom from recurrent IE 1 year 90% 5 years 78% 8 years 67%
	Martin Vobornik [53]	20 patients Commando procedure (4 patients) Hemi-Commando procedure (16 patients)	Mean follow-up 36 ± 31 months	Overall survival 1 year 60% 3 years 50% 5 years 45%		Freedom from reoperation 1 year 86% 3 years 71% 5 years 71%	1 patient (7.1%)
Commando vs DVR	Mona Kakavand [45]	129 Commando procedures 1191 DVR	Median follow-up Commando group 1.5 years DVR group 1.9 years	Operative deaths Entire Commando Group 14 (11%) DVR group 87 (7.3%)		Freedom from reoperation Commando group 5 years 87% DVR group 5 years 100%	
				Commando Group 30 days 92% 1 year 78% 3 years 66% 5 years 54%	DVR group 30 days 95% 1 year 86% 3 years 76% 5 years 67%		

Patients undergoing Commando surgery typically present with extensive annular abscess formation, involvement or destruction of AMC, fistula formation, or structural discontinuity between the aortic and mitral annuli. In contrast, standard DVR is generally performed in patients with preserved IVFB integrity and relatively limited tissue destruction. This fundamental difference in anatomical severity has a decisive impact on perioperative risk.

Accordingly, the higher operative mortality observed in Commando cohorts should not be attributed solely to procedural complexity, but rather reflects the underlying disease burden and preoperative hemodynamic instability [44]. Retrospective analyses indicate that, after adjustment for baseline clinical risk factors and disease severity, long-term survival may be comparable between the Commando and DVR groups, with the timing of surgical intervention serving as an additional determinant of outcome [41]. Furthermore, accumulating evidence suggests that, prior to risk adjustment, short-term complications following Commando reconstruction, including stroke, permanent pacemaker implantation, new-onset dialysis, and reoperation for bleeding, do not significantly differ from those observed in DVR cohorts [41]. Similarly, several studies have reported no statistically significant differences in long-term risk profiles or five-year survival between the two groups [45,47,54].

Overall, the Commando procedure should be regarded as a reconstructive strategy reserved for patients with severe infective endocarditis. It is important to acknowledge that the current evidence is largely derived from single-center retrospective studies with limited sample sizes and methodological heterogeneity in surgical techniques, timing of interventions, and perioperative management. Therefore, outcome data must be interpreted within the context of patient selection and baseline anatomical severity. Future multicenter collaborative registries and standardized reporting of outcome data are warranted to better delineate procedural risk independent of disease burden and to further refine appropriate surgical indications.

## 4. Perioperative Management

Patients undergoing the Commando procedure usually have aggressive IE, which requires radical debridement and complex reconstruction of both valves and the fibrous skeleton, which is more invasive and is associated with higher perioperative risks of bleeding, residual infection, and conduction system injury.

Careful perioperative management is therefore essential to improve outcomes in this high-risk population. Studies have emphasized the role of a multidisciplinary team involving cardiac surgery, infectious diseases, neurology/neurosurgery, and the intensive care unit to optimize perioperative outcomes [55,56]. However, data specifically addressing perioperative management strategies for the Commando procedure remain limited, and current practice largely relies on general IE management principles or experience with complex aortic root infections.

### 4.1 Antibiotic Therapy

The treatment of IE relies on complete surgical debridement and adequate antimicrobial therapy. For patients with active IE who undergo surgery, intravenous antibiotics are generally continued for about six weeks [13]. The duration is usually counted from the day of surgery or from the time the infection is considered controlled, and should be adjusted according to multiple factors such as the causative organism, antimicrobial susceptibility, intraoperative findings, and clinical response.

In cases of double-valve IE, early surgical intervention is often important for infection control. As a result, patients requiring a Commando procedure are frequently operated on during the active phase of infection [47]. When surgery is performed before full infection control is achieved, and intraoperative findings show extensive tissue destruction, aortic root abscess, fistula formation, or prosthetic infection, clinicians may choose to extend the course of antibiotic therapy.

Since many patients undergoing the Commando procedure have aortic root abscess or widespread structural involvement, the risk of residual infection may be higher. Therefore, after multidisciplinary discussion, prolonging antibiotic treatment or broadening antimicrobial coverage is reasonable. Postoperatively, close monitoring of inflammatory markers, repeated blood cultures, and echocardiographic assessment is important to identify recurrent infection or patch-related infection at an early stage.

### 4.2 Anticoagulation Strategies

Recent guidelines and reviews consistently state that IE itself is not an indication for anticoagulation. Anticoagulant therapy does not reduce the risk of infection and may increase the risk of hemorrhagic stroke [13,57]. The decision to initiate or continue anticoagulation should therefore be based on established indications, such as mechanical valve replacement, atrial fibrillation, or prior thromboembolic events, and should be reassessed in light of neurological complications and the risk of postoperative bleeding. In patients undergoing the Commando procedure, extensive patch reconstruction, complex root surgery, and a higher likelihood of reoperation contribute to a substantially increased perioperative bleeding risk.

For patients at very high thrombotic risk (e.g., mechanical valves, prior thromboembolism, high CHA_2_DS_2_-VASc score with atrial fibrillation), short-acting anticoagulation such as intravenous heparin may be considered once hemostasis is obtained, followed by transition to a vitamin K antagonist (VKA). In contrast, in patients with lower thrombotic risk, a more conservative approach is appropriate, prioritizing stable hemostasis and infection control and avoiding unnecessary bridging [4].

When cerebrovascular events occur, anticoagulation must be individualized. In patients with hemorrhagic stroke, significant hemorrhagic transformation, or risk of ruptured intracranial aneurysm, anticoagulation should be withheld, and the intracranial risk should be first addressed. In ischemic stroke without hemorrhagic transformation, anticoagulation is usually restarted in a delayed fashion, guided by repeat neuroimaging [4,57].

Overall, patients undergoing the Commando procedure often face both elevated thrombotic and bleeding risks. The intensity of anticoagulation should be tailored according to valve position, prosthesis type, and additional risk factors. For example, in patients with a mechanical aortic valve, the target international normalized ratio (INR) may be 2.5–3.5 in the presence of additional pro-thrombotic factors (e.g., hypercoagulable state, LVEF <35%, significant mitral stenosis with atrial fibrillation, or a major thrombotic event within the past 12 months), and 2.0–3.0 in their absence [58]. All decisions should be made within a multidisciplinary team framework.

### 4.3 Conduction Disturbances

IE is closely associated with atrioventricular (AV) conduction abnormalities, particularly in cases involving the aortic valve. Infection frequently extends beyond the valve leaflets to the annulus and adjacent structures, including the IVFB and membranous septum, predisposing patients to conduction system injury. Radical debridement of infected and necrotic tissue, together with annular reconstruction and patch repair, may further increase the risk of postoperative conduction block. The presence of an aortic root abscess itself is a risk factor for high-grade AV block [55,56].

Reported rates of permanent pacemaker (PPM) implantation after valve surgery for IE range from 7% to 15%, with higher rates observed in patients with extensive infection or prosthetic valve involvement [55,56]. In cohorts undergoing IVFB reconstruction with Commando variants, PPM implantation occurred in 20.8% of patients without root involvement and 30.0% of those with root involvement [48]. In aortic valve IE, high-grade AV block has been reported in 10–20% of cases [59]. Patients requiring PPM implantation more frequently underwent aortic root replacement and concomitant procedures, indicating more advanced disease at presentation [55,56]. Overall, conduction disturbances appear to reflect both the extent of infection and the severity of local inflammatory destruction [59].

Patients at high risk for conduction disturbances may benefit from early surgical intervention, close hemodynamic and rhythm monitoring in the intensive care unit, and timely temporary pacing support. The Commando procedure patients represent a particularly high-risk subgroup in which a predefined pacing strategy should be considered, including preoperative identification of high-risk imaging features (e.g., abscess adjacent to the membranous septum) and extensive debridement. Postoperatively, a lower threshold for maintaining temporary pacing and for evaluating the need for PPM implantation is reasonable, while carefully balancing infection control and the timing of device placement. When PPM implantation is required in the setting of IE, strict aseptic technique and appropriate antimicrobial coverage are essential. Suspected device-related infection should be managed according to established cardiovascular implantable electronic device-related infective endocarditis (CIED-IE) pathways [4].

Technical modifications may also influence the risk of conduction injury. In selected patients with small annuli of multiple valves, a Y-incision aortic annular enlargement combined with chimney mitral valve replacement has been proposed as an alternative to the traditional Commando procedure. By avoiding extensive division and reconstruction of the fibrous trigone, this approach may reduce the incidence of postoperative arrhythmias and PPM implantation, and may shorten ischemic and cardiopulmonary bypass times, thereby potentially lowering perioperative morbidity [60,61].

In summary, the Commando population represents one of the highest-risk phenotypes of aggressive double-valve infective endocarditis. Perioperative management is substantially more complex than in standard valve replacement, and considerable room remains for further refinement and investigation.

## 5. Discussion

Significant changes have occurred in epidemiology and microbiology of IE in recent years. Yet IE remains a long-standing clinical challenge and a significant economic burden, highlighting the need for individualized and comprehensive management. The treatment of IE requires a combination of prolonged antibiotic therapy and valve surgery, whose timing and method should be carefully determined by evaluating both the microbial characteristics and the extent of valve destruction.

This study reviewed the Commando and its modified procedures and their outcomes for complex IE involving destruction of the AMC. However, existing studies are limited, and long-term follow-up remains necessary to form more complete recommendations for the management of complex IE. Differences in disease progression should be considered when evaluating the outcomes of the different surgical procedures.

Indications for the Commando procedure also include severe calcification or prior radiation-induced cardiac damage requiring radical excision and anatomical reconstruction for valve implantation, and when standard DVR may pose a risk of LVOT obstruction, and annular enlargement is required in patients with small annuli.

For example, Tao and his team used the “Chimney” Commando procedure in patients with small aortic and mitral annuli [61].

**Fig. 4.**
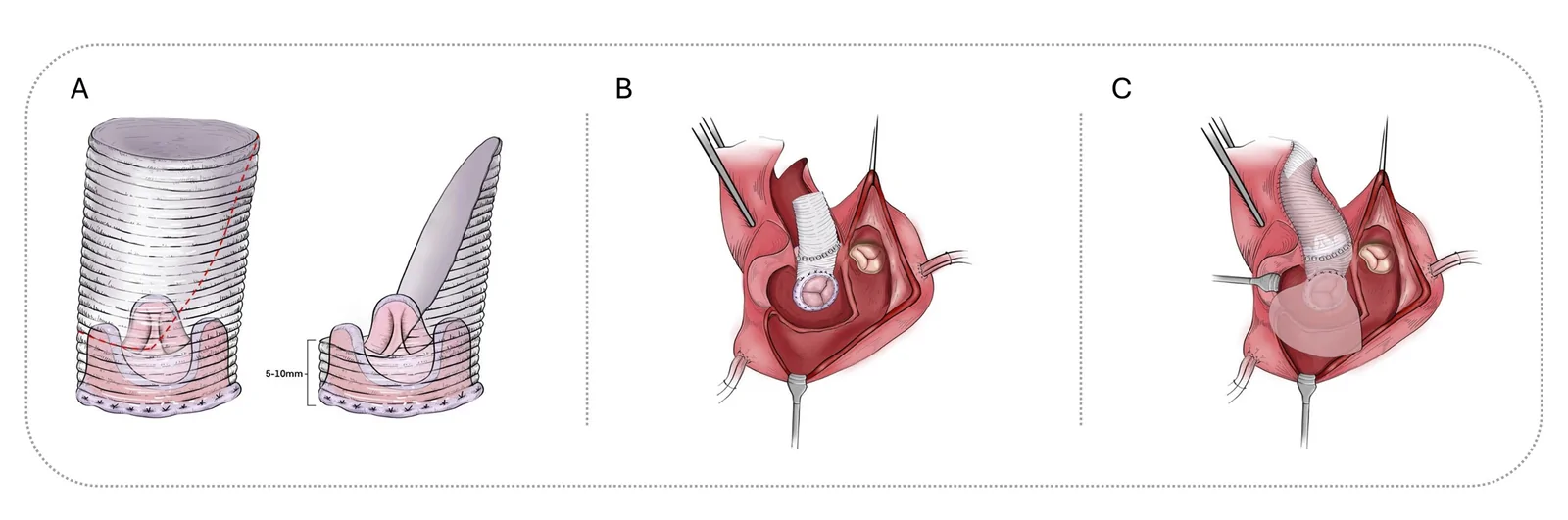
**“Chimney” Commando Procedure**. (A) The valve is secured within the vascular graft, with approximately three-quarters of the circumference of the self-assembled valvular conduit left as a free margin of 5–10 mm above the valve. (B) The valved conduit is anchored to the posterior mitral annulus or the endocardium of the posterior left atrial wall, while the aortic prosthesis is secured to both the annulus and the vascular patch. (C) The aortotomy is closed by the vascular patch, while the remaining patch of the mitral annular reconstruction is used to close the left atrial dome and interatrial septum.

In this procedure, the prosthetic mitral valve is secured inside the vascular graft using continuous 4-0 polypropylene sutures. Approximately three-quarters of the circumference of the self-assembled valvular conduit is left with a free margin of about 5–10 mm above the valve (Fig 4A).

Three-quarters of the circumference of the new mitral valve conduit is anchored to the native annulus, while the remainder attaches to the annulus reconstructed with the bovine pericardial patch (Fig. 4B). The trimmed vascular patch is used to fill the aortic annulus and the proximal defect of the ascending aorta, making it part of the aortic annulus, while closing the aortic incision, and the remaining patch is trimmed and used to close the top of the left atrium (Fig. 4C).

This procedure provides good hemodynamics and avoids LVOT obstruction with a better adjustment of the relative position and orientation between the mitral and the aortic prostheses [52], but still requires long-term outcomes data.

And Kakavand’s study [45] shows that there is no significant difference in cardiopulmonary bypass time, comorbidities, short-term outcomes and mortality rate between patients who received the Commando procedure and the standard DVR surgery. However, the Commando procedure may have a preference in small annulus patients [45].

## 6. Conclusion

Despite high perioperative and short-term mortality rates, the Commando procedure represents the only viable surgical option for survival for patients with double-valve IE affecting the AMC structure and offers better long-term survival outcomes. However, the current evidence of the Commando procedure remains limited. Most available studies consist of small retrospective series with limited patient numbers and relatively short follow-up durations, restricting robust evaluation of long-term outcomes. Although various modified Commando techniques have been proposed to simplify reconstruction and improve surgical feasibility, their long-term prognostic impact remains insufficiently defined. In addition, clear stratification of surgical indications and contraindications has not yet been established. Decisions regarding the use of conventional DVR, Hemi-Commando, or Commando reconstruction are still largely guided by anatomical findings and individual surgical experience. Perioperative management strategies also remain heterogeneous, and a standardized framework has yet to be developed. In order to achieve broader application of the Commando procedure, future studies based on multicenter collaboration, larger patient cohorts, and longer follow-up will be essential to better define surgical indications, evaluate reconstructive durability, and optimize perioperative management to improve outcomes.
